# The role of the right inferior frontal gyrus: inhibition and attentional control

**DOI:** 10.1016/j.neuroimage.2009.12.109

**Published:** 2010-04-15

**Authors:** Adam Hampshire, Samuel R. Chamberlain, Martin M. Monti, John Duncan, Adrian M. Owen

**Affiliations:** a Medical Research Council Cognition and Brain Sciences Unit, Chaucer Road, Cambridge, UK; b Department of Psychiatry, University of Cambridge School of Clinical Medicine, Addenbrooke's Hospital, Cambridge, UK

## Abstract

There is growing interest regarding the role of the right inferior frontal gyrus (RIFG) during a particular form of executive control referred to as response inhibition. However, tasks used to examine neural activity at the point of response inhibition have rarely controlled for the potentially confounding effects of attentional demand. In particular, it is unclear whether the RIFG is specifically involved in inhibitory control, or is involved more generally in the detection of salient or task relevant cues. The current fMRI study sought to clarify the role of the RIFG in executive control by holding the stimulus conditions of one of the most popular response inhibition tasks–the Stop Signal Task–constant, whilst varying the response that was required on reception of the stop signal cue. Our results reveal that the RIFG is recruited when important cues are detected, regardless of whether that detection is followed by the inhibition of a motor response, the generation of a motor response, or no external response at all.

## Introduction

Response inhibition can broadly be defined as the process by which a pre-potent, routine, or dominant response is deliberately withheld. Tasks that examine response inhibition typically involve the development of a routine response, followed by the effortful cancellation of that response when an infrequent stop cue is detected. This type of task manipulation is exemplified by the go/no-go task (GNG), the Stop Signal Task (SST), and their analogues (Logan and Cowan, 1984, Rubia et al., 2003, Aron et al., 2004). Results from these paradigms have lent considerable weight to the hypothesis that the right inferior frontal gyrus (RIFG) plays an important role in response inhibition. Most notably, fMRI research has revealed that the blood oxygenation level dependent (BOLD) signal within the RIFG increases at the point of inhibitory control when compared to a baseline of routine responding (Menon et al., 2001, Rubia et al., 2003, Aron et al., 2004). Furthermore, patients with frontal lobe lesions that include the RIFG are impaired on inhibitory control tasks (Aron et al., 2003b, 2007) whilst the selective noradrenalin reuptake inhibitor atomoxetine modulates RIFG metabolism and SST performance (Aron et al., 2003a, Chamberlain et al., 2009). It has been suggested that “there is a centrally located inhibitory mechanism” (van Boxtel et al., 2001), which “suppresses irrelevant responses” and that “inhibition is localized to right IFG alone” (Aron et al., 2004).

**Fig. 1:**
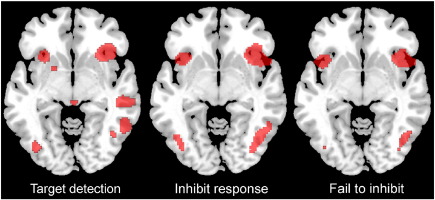
Activation associated with target detection and response inhibition. Fig. 1 illustrates the similar pattern of activation observed during target detection, and during both successful and failed inhibition in the SST task. Significant clusters are rendered in a region between the inferior frontal gyrus and anterior insula in all three conditions. Target detection data are taken from (Hampshire et al., 2007) whereas SST data are taken from combined published and unpublished data sets including (Chamberlain et al., 2009).

A potential limitation of the above account is that the GNG and SST tasks confound inhibitory control with the detection of a cue to stop, and it is unclear, therefore, whether the RIFG is specifically involved in generating inhibitory outputs, or plays a broader role in target detection. In favour of the more general account, the RIFG has been implicated in a range of other task demands (Duncan and Owen, 2000, Miller and Cohen, 2001), some of which have no obvious inhibitory component (Hampshire et al., 2009) and in some cases no overt task whatsoever (Hon et al., 2006). The most relevant example of this is the pattern of activation observed when pre-learnt target objects are detected (Linden et al., 1999, Hampshire et al., 2007, Hampshire et al., 2008b), a pattern similar to that observed during response inhibition (Fig. 1). Furthermore, a number of recent papers have proposed that the pattern of BOLD response during response inhibition is task dependent (Simmonds et al., 2008), whilst the IFG is recruited across a range of task conditions that require sustained attention and which have no obvious response inhibition component (Shallice et al., 2008a, Shallice et al., 2008b). On the basis of results from the broader literature and these latter findings, we suggest that the RIFG plays a general role in attentional control, rapidly adapting (Dehaene et al., 1998, Duncan, 2001) in order to respond to currently relevant and salient stimuli (Corbetta and Shulman, 2002) with inhibitory control in the GNG and SST being one particular instance of this process.

The aim of this study was to use an adapted version of the SST paradigm to test the inhibitory control and attentional control hypotheses of RIFG function. The former hypothesis predicted that the RIFG would only be recruited under increased inhibitory demand, i.e., when frequent and dominant responses are withheld, whereas the latter hypothesis predicted that the RIFG would be recruited whenever an important cue was detected regardless of the subsequent response and despite the increased difficulty associated with response inhibition.

## Materials and methods

### Task design

The task consisted of three blocks of scanning acquisition, each representing a variation on the classic SST design (Logan and Cowan, 1984, Rubia et al., 2003). The original task design is described in detail elsewhere (Logan and Cowan, 1984). In general, across all three blocks, participants viewed a series of left and right pointing arrows that appeared on-screen in rapid succession. Occasionally, an up arrow appeared a short variable delay after the onset of the left or right arrow and this formed the cue for an additional behaviour that varied across the three blocks.

During the first block, participants were instructed to silently count the total number of up arrow cues observed over the block, without making any motor responses to these targets (“COUNT”). At the end of the block, participants were asked to report verbally the total number of up arrows that they had detected. This condition allowed us to examine whether RIFG BOLD signal increases were elicited during target detection without an overt motor responses.

In the second block, participants responded to the up-arrow cue with a left or right button press according to the immediately preceding lateral arrow. This condition was intended to examine whether the BOLD signal in the RIFG increased when cue detection was associated with the generation of a motor response (as opposed to the cancellation of a motor response in the classical SST design) (“RESPOND”).

In the third acquisition block, participants were instructed to make left or right button presses after the appearance of left/right arrows, but to withhold responses whenever an up arrow occurred (“INHIBIT”). This condition was therefore equivalent to the response inhibition manipulation employed in the classical SST design.

There was a short pre-training session in which the participants were briefly instructed of the task demands and they were also reminded of the current instructions verbally before each block began. Importantly, the participants did not undertake the tasks themselves, but were merely instructed as to the conditions that they would undertake. There was, therefore, no opportunity to develop an association between the up arrow cue and the inhibition of a response. For similar reasons, the block order was always fixed with the INHIBIT block last in order to avoid the potential confound of up arrows being associated with inhibition during the other two conditions. Counterbalancing for order was unnecessary as the hypotheses under examination–i.e., that the RIFG is recruited due to cue detection in general as opposed to response inhibition in particular–was dependent upon identifying significant BOLD response to up arrows in all three acquisition blocks as opposed to a direct contrast between blocks.

Participants viewed a total of 131 left and 131 right arrows per 9-min acquisition block, 68 of which were followed by up arrows. Left and right arrows were displayed on the screen for 300 ms with a predefined pseudo-randomised ISI such that arrows occurred at either 1600, 1700, 1800, 1900, or 2000 ms intervals. Up arrows were displayed unpredictably after the left and right arrows with a randomised offset from the start of the left or right signal of 300 to 900 ms. This offset was chosen because it encompasses a similar range to that previously reported for the SST, for example (Rubia et al., 2003) reported an offset of 678 ms at 50% failure. This time gap was not varied dynamically to balance for the frequency of successful vs. unsuccessful inhibition (Williams et al., 1999) as this would not have been possible for the counting and motor response control conditions.

### Scanning acquisition

Fourteen right handed participants undertook the fMRI task at the MRC Cognition and Brain Sciences Unit using a 3 Tesla Siemens Tim Trio scanner. 310 T2-weighted echo-planar images depicting BOLD contrast were acquired per block of scanning acquisition, with the first 10 discarded to avoid T1 equilibrium effects. Each image consisted of 32 ⁎ 3 mm slices (1 mm inter-slice gap, descending slice order) each with a 64 × 64 matrix, a 192 × 192 mm field of view. Images were collected with a 2-s repetition time, a TE of 30 ms, a flip angle of 78°, echo spacing of 0.51 ms, and a bandwidth of 2232 Hz/Px. The experiment was programmed in Visual Basic 6 and the display projected onto a screen, visible from the scanner via a mirror. Responses were made on a custom button box using the first two fingers of the right hand.

### Imaging analysis

Images were pre-processed and analysed using the Statistical Parametric Mapping 5 software (SPM5, Wellcome Department of Cognitive Neurology). Images were slice time corrected, reoriented to correct for subject motion, spatially normalised to the standard Montreal Neurological Institute (MNI) template, smoothed with an 8 mm full-width at half-maximum Gaussian kernel, and high-pass filtered prior to analysis (cutoff period 180 s).

Fixed effects analyses were carried out on each participant's data using general linear models in SPM5. Each acquisition block was modeled according to two regressors, the first being the onsets of all up arrow cues convolved with the canonical haemodynamic response function, and the second being the constant of the regression model. The routine left and right arrows were left intrinsic in the constant term as the low temporal resolution of the haemodynamic response function meant that they could not be estimated separately–consistent with previous studies (Rubia et al., 2003). The regressors were, therefore, identical across the three acquisition blocks in order to maximise cross-block comparability.

Note that, in the RESPOND block, it could be argued that response to left and right arrows must be withheld until detection of the up arrow cue, potentially a form of inhibition. In this case, however, inhibitory activation would occur at all trials and would be modeled by the baseline regressor, not the up arrow cue regressor.

**Fig. 2:**
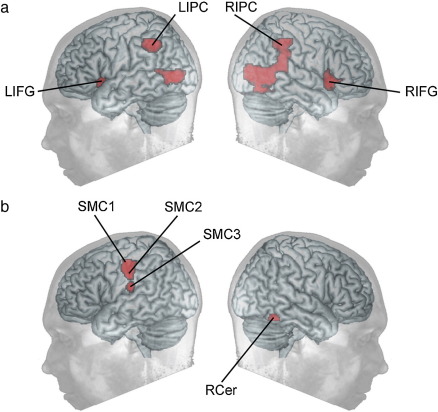
ROIs defined on the basis of previously acquired SST data. Fig. 2 illustrates the regions of interest (ROIs) generated from the analysis of 81 participants who previously undertook the fMRI Stop Signal Task. (A) ROIs were rendered bilaterally in the inferior frontal gyri (LIFG & RIFG) and in the inferior parietal cortex (IPC) from the contrast of inhibition minus baseline. (B) Contrasting failed minus successful inhibition rendered ROIs in three locations in the left sensorimotor cortex (SMC1, SMC2, & SMC3) and in the right cerebellum (RCer).

Regions of interest (ROIs) were defined using the MarsBaR ROI toolbox (Brett et al., 2002). This approach enabled us to focus on those brain regions that were of maximal importance to the hypotheses under investigation. ROIs were generated orthogonally using previously collected data in which 81 participants (Chamberlain et al., 2009 and unpublished data) undertook an identical SST fMRI paradigm to that reported by Rubia et al. (2003). Peak activation clusters were generated from this large dataset using SPM5, with a threshold of p = 0.05 FWE corrected for the whole brain mass and a 50 voxel extent threshold, for two main contrasts. Inhibition related ROIs were identified using the contrast of successful inhibition versus baseline responding to go trials (Fig. 2A). In line with previous findings (Rubia et al., 2003) these involved clusters for bilateral IFG (all coordinates in MNI space) (BA13, BA47, BA45, LIFG x = −36, y = 16, z = −4; RIFG x = 42, y = 18, z = −6), and the bilateral inferior parietal cortex (BA 40, LIPC x = −50, y = −48, z = 44; RIPC x = 50, y = −42, z = 48). It has recently been suggested that the pre supplementary motor area (preSMA) is involved in response inhibition (Li et al., 2006) a region that was also activated in the inhibition vs. baseline contrast, and for this reason an ROI was generated in the preSMA (BA6, x = 0, y = 22, z = 46). We also generated ROIs from the contrast of failed minus successful inhibition in order to isolate the network involved in generating motor responses. This yielded ROIs in the right cerebellum (x = 18, y = −52, z =−20), and in a swathe of left sensorimotor cortex (SMC) (BA3 BA4 BA6 & BA 43, peak coordinates from dorsal to ventral SMC1 x = −42, y = −20, z = 52; SMC2 x = −54, y = −18, z = 42; SMC3 x = −54, y = −20, z = 18) (Fig. 2B)–consistent with the right-handed response employed in the task. The right cerebellum (RCer), and the SMC3 cluster were used to generate ROIs, whereas two 5 mm radius spheres were generated at the peak coordinates for SMC1 and SMC2 due to the clusters being close and contiguous. One influential model has proposed that interactions between the RIFG and the subthalamic nucleus (STN) are critical in response inhibition (Aron and Poldrack, 2006). This region was not significantly activated for the contrast of successful minus unsuccessful inhibition in our previously collected data set. Consequently, 5 mm radius spherical ROIs were defined based on previously reported coordinates (Aron and Poldrack, 2006) in the STN bilaterally (x = +/−10, y = −15, z = −5). These coordinates are central to the STN in the MNI template (Prodoehl et al., 2008).

The above-defined ROIs were then used to investigate BOLD responses in the current study for the three blocks of interest (COUNT, RESPOND, INHIBIT). Specifically, β values for the up arrow regressors were averaged across all voxels within these ROIs for the three acquisition blocks using the MarsBar ROI toolbox and these data were exported for group-level random effects analyses in SPSS. Significant BOLD responses in each ROI were identified in a series of one sample *t* tests and analysis of variance (ANOVA) was used to identify effects of block type and hemisphere.

Group level random effects analysis was also carried out at the voxel level unconstrained within the whole brain volume in a full factorial design in SPM 5 in which the within subject factor was acquisition block. Results from this analysis were corrected for multiple comparisons across the whole brain mass using FWE correction at p < 0.05.

## Results

### Behavioural results

The relative difficulty of the three task blocks was gauged by total errors made. In the first block, error rate was taken as the difference between the number of up arrows displayed and the number reported. In the second block, the score was the number of up arrow cues that were not followed by a correct button press, whilst in the third block it was the number of instances in which the participant failed to withhold a button press.

On average, participants made 1.86 errors when counting in the first block, 0.86 when responding in the second block, and 13.93 when attempting to withhold responses in the third block. Differences between block 1 and block 3 (t = 3.46; p < 0.005) and between block 2 and block 3 (t = 3.99; p < 0.005) were both significant. The difference between block 1 and block 2 was not significant (p > 0.1). Overall, these results indicate that the behaviour associated with cues in the INHIBIT condition was more difficult than for the COUNT or the RESPOND conditions.

### Imaging results

**Fig. 3:**
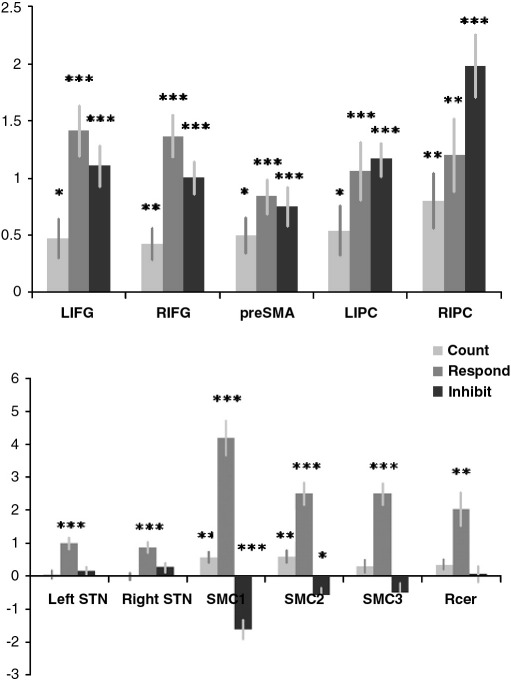
Results from the ROI analysis. Fig. 3 illustrates the results from the main ROI analyses. The IFG bilaterally, and the left inferior parietal cortex showed significant increases in the BOLD response when the up arrow cue was detected in all three acquisition blocks. By contrast, the motor related ROIs all showed increased BOLD signal to up arrow cues selectively when the subsequent response was a button press. Interestingly, the SMC1 and SMC2 ROIs were also significantly deactivated when the subsequent response was the inhibition of a button press. ⁎ p < 0.05, ⁎⁎ p < 0.01, ⁎⁎⁎ p < 0.001. The Y axis is regressor β weight and error bars show the standard error of the mean.

Fig. 3 indicates mean BOLD responses in each ROI for each of the three block types (COUNT, RESPOND, INHIBIT). The one sampled *t* tests for positive values of the target (up arrow) regressor showed significant increases in BOLD signal in the bilateral IFG, the preSMA and the IPC during all three blocks. BOLD responses in the SMC3 and RCer ROIs occurred only during RESPOND as predicted. The SMC1 and SMC2 ROIs showed a more complex profile, being significantly activated during COUNT and RESPOND, and significantly deactivated during INHIBIT. The STN ROIs showed no significant increase in BOLD signal to cues during COUNT, a sub-threshold trend towards an increase in the right hemisphere during INHIBIT, and a much greater increase bilaterally during RESPOND.

There was a main effect of block type on IFG BOLD activation (F_(1,13)_ = 10.75; p < 0.01) but there was no significant main effect of hemisphere, nor was there a significant interaction between block and hemisphere. Paired *t* tests indicated significantly greater activation in the left and right IFG during RESPOND and INHIBIT blocks compared to COUNT blocks. Importantly, there was no significant difference in left or right IFG activation between RESPOND and INHIBIT blocks despite the fact that the INHIBIT condition was behaviourally far more difficult. There was no main effect of block type on BOLD activation in the IPC and no interaction between block and hemisphere. There was, however, a main effect of hemisphere favouring heightened BOLD response to up arrow cues in the right IPC (F_(1,13)_ = 19.63; p < 0.001). There was no main effect of block in the preSMA. There was a large main effect of block in the STN (F_(1,13)_ = 34.06; p < 0.001) and no significant interactions.

In some previous studies, the reception of up arrow cues has been broken down into two separate regressors depending on whether or not they resulted in successful cancellation of the motor response. It has previously been reported that the contrast of these two regressors resulted in increased activation during trials where the participant successfully inhibits the response compared with failure to inhibit (Rubia et al., 2003). We therefore also examined the third acquisition block modeling separately for successful and unsuccessful response inhibition. Interestingly, comparison of the successful and failed inhibition regressors rendered the reverse result in the IFG and the preSMA ROIs in paired sample tests (LIFG t = −3.278; p < 0.01; RIFG t = −2.464; p < 0.05; preSMA t = −2.603; p = 0.05), with increased activation during failed inhibition, a result that has been reported previously (Menon et al., 2001) and which, in this instance, may be attributable to the lowered frequency of failed inhibition trials causing them to be more salient (Braver et al., 2001).

**Fig. 4:**
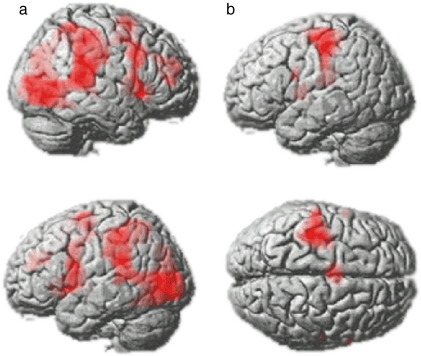
Results from the whole brain analysis. (A) The whole brain analysis revealed significant increases in BOLD signal when up arrow cues were detected across all three task conditions in a network of brain regions including the IFG bilaterally. There was no significant difference between acquisition blocks in the RIFG even at a liberal uncorrected threshold. (B) There was a significant effect of block within the left sensorimotor cortex ROIs.

When collapsed across block, the whole brain analysis showed significantly increased BOLD response to up arrows cues across a broad swathe of frontal and parietal cortex including the IFG and the preSMA (Fig. 4A). There was also a significant main effect of acquisition block in the left sensorimotor cortex and the right cerebellum (Fig. 4B). There were no significant voxels for the main effect of acquisition block within the RIFG.

Peak activation foci used to define the RIFG ROI were rather posterior and medial, and spread from the right anterior insula across the inferior frontal operculum. Whilst these coordinates were highly similar to the peak activation foci previously published for the SST (Rubia et al., 2003, Aron et al., 2004) and for target detection (Linden et al., 1999, Hampshire et al., 2007, Hampshire et al., 2008b) more lateral and anterior portions of the RIFG were not encapsulated within this ROI. FWE whole brain correction is conservative. To rule out the possibility that other portions of the RIFG were particularly involved in inhibitory control, but were below the corrected threshold, we examined the whole brain at the voxel level for the contrast of stop minus go (block 3 minus block 2) with the reduced threshold of p = 0.001 uncorrected for the whole brain mass. No significant voxels were rendered in the IFG even at this low threshold, although one small cluster (12 voxels) was observed in the superior frontal gyrus (x = −10, y = 36, z = 46).

## Discussion

The results presented here accord well with those previous studies that have reported a role for the RIFG in the inhibition of pre-potent responses during the GNG and SST paradigms (Menon et al., 2001, van Boxtel et al., 2001, Aron et al., 2003a, Aron et al., 2003b, Rubia et al., 2003, Aron et al., 2004, Picton et al., 2007, Verbruggen and Logan, 2008). However, we found no evidence to support the hypothesis that the RIFG plays a unique or specialised role in inhibition and furthermore, the data run counter to this hypothesis in a number of key respects.

Firstly, if the RIFG were involved in inhibitory control specifically, then the BOLD response should have increased specifically during the inhibition of a pre-potent response. Instead, the counting of cues, the initiation of responses, and the inhibition of responses all activated the RIFG. Thus, from a functional perspective, it is more parsimonious to state that the RIFG responds whenever salient cues that have a bearing on the current task plan are detected (Hampshire et al., 2009). Secondly, we found no evidence to support the idea that “inhibition is localized to the RIFG alone” (Aron et al., 2004), but instead, the inhibitory manipulation in the SST recruited a network of brain regions including the IFG bilaterally, the preSMA, and the IPC bilaterally. Importantly, these additional brain regions were also co-recruited during the COUNT and RESPOND conditions. Finally, we did not find evidence that the IFG interacts with the STN to suppress initiated motor responses (Aron and Poldrack, 2006) as no significant increase in BOLD response was observed in the INHIBIT condition. As the STN was highly active during the RESPOND condition, it seems probable that the lack of an observed effect during INHIBITION condition is due to the STN being involved to a similar extent during both motor generation and motor suppression in that block.

Whilst the results of the current study run counter to the hypothesis that the RIFG is specialised to inhibition and plays a unique role in inhibition alone, there are a number of more complex possibilities. For example, one could suggest that neurons within the RIFG were sub-divided into two distinct populations, one coding for task relevant cues, and the other for inhibitory outputs. This seems unlikely, as a disproportionate BOLD response to cues would have been predicted in the inhibition block compared with the two control conditions. Indeed, in the current task design we actively biased towards finding such a result, as the inhibitory condition was clearly a much more demanding manipulation. Instead, an increased response was seen during both response generation *and* response inhibition when compared with counting. Alternatively, one could argue that there were at least three distinct overlapping populations within the right IFG, one coding for cue detection, another coding for the generation of motor responses, and another coding for inhibitory control. The differential recruitment of these two additional populations could somehow conspire to mask the BOLD response of the inhibitory population. It becomes necessary, however, to complement this increasingly complex model with an increasing degree of specificity in order to explain the observed data. For example, as the BOLD response at response generation is equivalent to that at response suppression, the motor generation sub-population would have to be recruited only when responses were infrequent during RESPOND or to responses in general but to exactly half the extent as those coding for response inhibition.

Even this most complex and specified model cannot account for recent findings from the broader literature. For example, one recent study sought to examine the inhibitory control condition in the SST using functional connectivity (Duann et al., 2009). That study demonstrated in a large cohort that there is no evidence for a direct inhibitory influence from the RIFG on components of the motor system such as the STN and the pre motor cortex whilst undertaking the SST. Instead the RIFG appeared to exert its influence over the motor system via potentiating inputs to the preSMA.

As these examples show, to account for the broad range of circumstances in which the RIFG is active, the inhibitory hypothesis must postulate hidden inhibitory components for which often there is no direct evidence. A simpler hypothesis, which we suggest can offer a more parsimonious explanation of both the data presented here and its relationship to the broader literature on executive control, is that the RIFG, along with the left IFG, the PPC and the preSMA, form a network that rapidly tunes to represent those inputs and responses that form the currently intended task schema. Thus, the response within this network is particularly strong when cues are detected that trigger effortful and task relevant behaviours.

Research into the role of right lateral prefrontal cortex–a likely analogue of the RIFG in non-human primates (Petrides and Pandya, 2002)–offers clues regarding the probable neural mechanism by which this brain region exerts executive control. When single unit recordings are taken from this region, neurons display a highly adaptive profile, with a large proportion rapidly adapting to respond to the currently relevant stimuli, stimulus dimensions, and responses (Freedman et al., 2001, Miller and Cohen, 2001, Everling et al., 2002, Duncan, 2006). Also, neurons in this region that respond to task specific information continue to respond when that information is being actively maintained over a delay (Fuster and Alexander, 1971, Funahashi et al., 1989, Rao et al., 1997). Such maintenance activity may modulate stimulus processing in specialised regions of posterior cortex (Chelazzi et al., 1998, Kastner et al., 1999). Finally, many brain regions exhibit inhibition at a local level, with inputs competing for limited capacity processing resources (Duncan, 2006). Herein lies a potential key to the likely neural process by which inhibitory control is exerted. In terms of visual processing, inhibition of one object when attention is focused on another can be explained as a secondary effect, i.e., an emergent property of local competition when one competing item is subjected to top-down potentiating signals which have their source in the lateral prefrontal cortex (Norman and Shallice, 1980). Thus, when considering the case of selective attention in the face of increased distraction, it may well be the case that increased inhibition of distractors is achieved simply by actively focusing more willfully on that which is attended as opposed to directly suppressing that which is distracting. It seems reasonable to suggest that at an executive level stopping and going in the SST task are represented as two alternate behaviours. If these two representations are competing for processing resources, then focusing attention on one will tend to inhibit the other.

Accordingly, from a quantitative perspective, the response inhibition manipulation in the GNG and SST tasks is often conceived in terms of alternate stop and go processes that compete for the earliest completion time–the horse race model (Logan and Cowan, 1984, Band et al., 2003). If the routine go process executes before the infrequent stop process, then inhibition fails. Another perspective on this relationship is that the SST task schema is represented in two alternate action plans that compete to be executed. The stop plan is executed frequently and becomes routine and dominant, requiring minimal effort and minimal executive control from the IFG/PPC/preSMA network. Processing of the plan to stop, by comparison, is not routine and requires effortful monitoring for the stop cue and application of a top-down biasing signal in order to allow it to win the competition for execution. From the attentional tuning perspective, the contribution of the RIFG to inhibitory control can be considered akin, therefore, to that made when responding to or counting infrequent targets, i.e., the effortful maintenance and execution of a planned behaviour.

The relationship between potentiation and inhibition also becomes apparent when considering some of the other task manipulations that have previously been cited as evidence for a specific role for the RIFG in inhibitory control. For example, it has been noted that when suppressing intrusive thoughts, activity within the RIFG increases and that the RIFG appears to interact with regions of the temporal lobe that are known to be crucial to memory (Anderson et al., 2004). On the surface, this appears to be good evidence that the RIFG is directly suppressing the representation of unwanted thoughts and memories within the temporal lobes. An alternative explanation for these findings, however, is that the RIFG is engaged in a coping strategy–for example retrieving an alternative thought or memory in order to swamp limited capacity processing resources. From a phenomenological perspective, this seems rather more likely, as when attempting not to think about something, an individual will typically try and think about something else, whilst experimentally, it is well established that trying to push a negative thought away directly can lead to increases in both the frequency and emotional impact of that thought (Wegner et al., 1987, Wegner, 1989, Purdon and Clark, 1999, Wenzlaff and Wegner, 2000).

This latter take on the inhibition of thoughts ties in rather closely with the well established role of the IFG in the deliberate formation and retrieval of information in long term memory (Dove et al., 2006). Previously, it has been suggested that the RIFG is recruited under these conditions as it becomes necessary to inhibit other memories when attempting to encode or retrieve a target memory (Aron et al., 2004). By contrast, potentiating some sub-portion of the neurons that form the memory, and then allowing the rest of the memory to activate via a process of pattern completion is far closer to the generally accepted view of active memory retrieval via cues (Henson et al., 1999). This interpretation is also analogous to the observation that the IFG is not just recruited during selective retrieval of semantic information, where inhibition of competing representations may be necessary, but also during the effortful retrieval of semantic information in general (Wagner et al., 2001).

Finally, there is strong evidence that the RIFG plays a role in attentional switching (Dove et al., 2000, Cools et al., 2002, Hampshire and Owen, 2006)–the process by which the focus of attention is moved from one locus to another (Monsell, 2003). Again, on the surface, the inhibitory control hypothesis seems well able to account for the role played by the RIFG in attentional switching. The suggestion would be that the RIFG facilitates the attentional switch by inhibiting the previously attended object, location, or dimension, thereby allowing attention to shift away. It could be predicted from this account, that when switching attention away from a previously rewarded and routine response, for example during reversal learning, a much greater degree of inhibition should be necessary in order to overcome the pre-potent response. Whilst it is the case that the RIFG is recruited during reversal learning (Cools et al., 2002) it has recently been reported that (unlike the lateral orbitofrontal cortex) activation within the RIFG is no greater at the point of a reversal than when switches are carried out between previously unrewarded objects, none of which can be considered pre-potent (Hampshire and Owen, 2006, Chaudhry et al., Under Submission). To complicate the issue further, the extent to which the RIFG is recruited during attentional switching does appear to relate to the visual difference between the current and previous stimuli–so whilst switches of attention between similar objects recruit the RIFG more than non-switches, switches between objects drawn from different categories recruit RIFG to an even greater extent (Hampshire and Owen, 2006, Hampshire et al., 2008a). Overall these findings accord best with a role for the RIFG in reconfiguring a representation of the currently attended input–a role that may be shared with other regions of the frontoparietal network including the IPC.

It undoubtedly remains the case that the GNG and SST paradigms are robust markers of RIFG function and in this respect they provide powerful tools for investigating the neural basis of executive dysfunction (Rubia et al., 1999, Aron et al., 2003b) and measuring the efficacy of pharmacological interventions (Aron et al., 2003a, Chamberlain et al., 2009). Also, one cannot entirely rule out the possibility that a sub-population of neurons within the RIFG work to exert an inhibitory influence over processes within other brain regions. However, what is clear is that the IFG plays a more general role in executive function than just the exertion of inhibitory control. Thus, the results from the tasks such as the GNG and the SST should not be over interpreted in terms of neural inhibition. For the future, a pertinent question is whether patient groups that perform poorly on GNG and SST tasks can be sub-divided according to whether the underlying impairment is an inability to maintain attention when looking for cues, or an inability to suppress a response when the cue is detected.

## References

[bib1] Anderson M.C., Ochsner K.N., Kuhl B., Cooper J., Robertson E., Gabrieli S.W., Glover G.H., Gabrieli J.D. (2004). Neural systems underlying the suppression of unwanted memories. Science.

[bib2] Aron A.R., Dowson J.H., Sahakian B.J., Robbins T.W. (2003). Methylphenidate improves response inhibition in adults with attention-deficit/hyperactivity disorder. Biol. Psychiatry.

[bib3] Aron A.R., Fletcher P.C., Bullmore E.T., Sahakian B.J., Robbins T.W. (2003). Stop-signal inhibition disrupted by damage to right inferior frontal gyrus in humans (vol 6, pg 115, 2003). Nat. Neurosci..

[bib4] Aron A.R., Poldrack R.A. (2006). Cortical and subcortical contributions to Stop signal response inhibition: role of the subthalamic nucleus. J. Neurosci..

[bib5] Aron A.R., Robbins T.W., Poldrack R.A. (2004). Inhibition and the right inferior frontal cortex. Trends Cogn. Sci..

[bib6] Band G.P.H., van der Molen M.W., Logan G.D. (2003). Horse-race model simulations of the stop-signal procedure. Acta Psychol..

[bib7] Braver T.S., Barch D.M., Gray J.R., Molfese D.L., Snyder A. (2001). Anterior cingulate cortex and response conflict: effects of frequency, inhibition and errors. Cereb. Cortex.

[bib8] Brett, M., Anton, J., Valabregue, R. and Poline, J., 2002. Region of interest analysis using an SPM toolbox [abstract]. Proceedings of the 8th International Conference on Functional Mapping of the Human Brain, Sendai, Japan, pp.

[bib9] Chamberlain S.R., Hampshire A., Muller U., Rubia K., Campo N.D., Craig K., Regenthal R., Suckling J., Roiser J.P., Grant J.E., Bullmore E.T., Robbins T.W., Sahakian B.J. (2009). Atomoxetine modulates right inferior frontal activation during inhibitory control: a pharmacological functional magnetic resonance imaging study. Biol. Psychiatry.

[bib10] Chaudhry, A., Hamphire, A., Owen, A. M. and Roberts, A. C., Under Submission. Differentiating the contribution of lateral orbitofrontal cortex and inferior frontal gyrus to reversal learning with functional MRI.

[bib11] Chelazzi L., Duncan J., Miller E.K., Desimone R. (1998). Responses of neurons in inferior temporal cortex during memory-guided visual search. J. Neurophysiol..

[bib12] Cools R., Clark L., Owen A.M., Robbins T.W. (2002). Defining the neural mechanisms of probabilistic reversal learning using event-related functional magnetic resonance imaging. J. Neurosci..

[bib13] Corbetta M., Shulman G.L. (2002). Control of goal-directed and stimulus-driven attention in the brain. Nat. Rev. Neurosci..

[bib14] Dehaene S., Kerszberg M., Changeux J.P. (1998). A neuronal model of a global workspace in effortful cognitive tasks. Proc. Natl. Acad. Sci. U. S. A..

[bib15] Dove A., Brett M., Cusack R., Owen A.M. (2006). Dissociable contributions of the mid-ventrolateral frontal cortex and the medial temporal lobe system to human memory. Neuroimage.

[bib16] Dove A., Pollmann S., Schubert T., Wiggins C.J., von Cramon D.Y. (2000). Prefrontal cortex activation in task switching: an event-related fMRI study. Cogn. Brain Res..

[bib17] Duann J.R., Ide J.S., Luo X., Li C.S. (2009). Functional connectivity delineates distinct roles of the inferior frontal cortex and presupplementary motor area in stop signal inhibition. J. Neurosci..

[bib18] Duncan J. (2001). An adaptive coding model of neural function in prefrontal cortex. Nat. Rev. Neurosci..

[bib19] Duncan J. (2006). EPS Mid-Career Award 2004: brain mechanisms of attention. Q. J. Exp. Psychol. (Colchester).

[bib20] Duncan J., Owen A.M. (2000). Common regions of the human frontal lobe recruited by diverse cognitive demands. Trends Neurosci..

[bib21] Everling S., Tinsley C.J., Gaffan D., Duncan J. (2002). Filtering of neural signals by focused attention in the monkey prefrontal cortex. Nat. Neurosci..

[bib22] Freedman D.J., Riesenhuber M., Poggio T., Miller E.K. (2001). Categorical representation of visual stimuli in the primate prefrontal cortex. Science.

[bib23] Funahashi S., Bruce C.J., Goldman-Rakic P.S. (1989). Mnemonic coding of visual space in the monkey's dorsolateral prefrontal cortex. J. Neurophysiol..

[bib24] Fuster J.M., Alexander G.E. (1971). Neuron activity related to short-term memory. Science.

[bib25] Hampshire A., Duncan J., Owen A.M. (2007). Selective tuning of the blood oxygenation level-dependent response during simple target detection dissociates human frontoparietal subregions. J. Neurosci..

[bib26] Hampshire A., Gruszka A., Fallon S.J., Owen A.M. (2008). Inefficiency in self-organized attentional switching in the normal aging population is associated with decreased activity in the ventrolateral prefrontal cortex. J. Cogn. Neurosci..

[bib27] Hampshire A., Owen A.M. (2006). Fractionating attentional control using event-related fMRI. Cerebral. Cortex.

[bib28] Hampshire A., Thompson R., Duncan J., Owen A.M. (2008). The target selective neural response–similarity, ambiguity, and learning effects. PLoS ONE.

[bib29] Hampshire A., Thompson R., Duncan J., Owen A.M. (2009). Selective tuning of the right inferior frontal gyrus during target detection. Cogn. Affect Behav. Neurosci..

[bib30] Henson R.N.A., Shallice T., Dolan R.J. (1999). Right prefrontal cortex and episodic memory retrieval: a functional MRI test of the monitoring hypothesis. Brain.

[bib31] Hon N., Epstein R.A., Owen A.M., Duncan J. (2006). Frontoparietal activity with minimal decision and control. J. Neurosci..

[bib32] Kastner S., Pinsk M.A., De Weerd P., Desimone R., Ungerleider L.G. (1999). Increased activity in human visual cortex during directed attention in the absence of visual stimulation. Neuron.

[bib33] Li C.S., Huang C., Constable R.T., Sinha R. (2006). Imaging response inhibition in a stop-signal task: neural correlates independent of signal monitoring and post-response processing. J. Neurosci..

[bib34] Linden D.E.J., Prvulovic D., Formisano E., Vollinger M., Zanella F.E., Goebel R., Dierks T. (1999). The functional neuroanatomy of target detection: an fMRI study of visual and auditory oddball tasks. Cereb. Cortex.

[bib35] Logan G.D., Cowan W.B. (1984). On the ability to inhibit thought and action–a theory of an act of control. Psychol. Rev..

[bib36] Menon V., Adleman N.E., White C.D., Glover G.H., Reiss A.L. (2001). Error-related brain activation during a Go/NoGo response inhibition task. Hum. Brain Mapp..

[bib37] Miller E.K., Cohen J.D. (2001). An integrative theory of prefrontal cortex function. Annu. Rev. Neurosci..

[bib38] Monsell S. (2003). Task switching. Trends. Cogn. Sci..

[bib39] Norman D.A., Shallice T., Davidson R. (1980). Attention to action: willed and automatic control of behaviour.

[bib40] Petrides M., Pandya D.N. (2002). Comparative cytoarchitectonic analysis of the human and the macaque ventrolateral prefrontal cortex and corticocortical connection patterns in the monkey. Eur. J. Neurosci..

[bib41] Picton T.W., Stuss D.T., Alexander M.P., Shallice T., Binns M.A., Gillingham S. (2007). Effects of focal frontal lesions on response inhibition. Cereb. Cortex.

[bib42] Prodoehl J., Yu H., Little D.M., Abraham I., Vaillancourt D.E. (2008). Region of interest template for the human basal ganglia: comparing EPI and standardized space approaches. Neuroimage.

[bib43] Purdon C., Clark D.A. (1999). Metacognition and obsessions. Clin. Psychol. Psychother..

[bib44] Rao S.C., Rainer G., Miller E.K. (1997). Integration of what and where in the primate prefrontal cortex. Science.

[bib45] Rubia K., Overmeyer S., Taylor E., Brammer M., Williams S.C., Simmons A., Bullmore E.T. (1999). Hypofrontality in attention deficit hyperactivity disorder during higher-order motor control: a study with functional MRI. Am. J. Psychiatry.

[bib46] Rubia K., Smith A.B., Brammer M.J., Taylor E. (2003). Right inferior prefrontal cortex mediates response inhibition while mesial prefrontal cortex is responsible for error detection. Neuroimage.

[bib47] Shallice T., Stuss D.T., Alexander M.P., Picton T.W., Derkzen D. (2008). The multiple dimensions of sustained attention. Cortex.

[bib48] Shallice T., Stuss D.T., Picton T.W., Alexander M.P., Gillingham S. (2008). Mapping task switching in frontal cortex through neuropsychological group studies. Front. Neurosci..

[bib49] Simmonds D.J., Pekar J.J., Mostofsky S.H. (2008). Meta-analysis of Go/No-go tasks demonstrating that fMRI activation associated with response inhibition is task-dependent. Neuropsychologia.

[bib50] van Boxtel G.J.M., van der Molen M.W., Jennings J.R., Brunia C.H.M. (2001). A psychophysiological analysis of inhibitory motor control in the stop-signal paradigm. Biol. Psychol..

[bib51] Verbruggen F., Logan G.D. (2008). Response inhibition in the stop-signal paradigm. Trends Cogn. Sci..

[bib52] Wagner A.D., Pare-Blagoev E.J., Clark J., Poldrack R.A. (2001). Recovering meaning: Left prefrontal cortex guides controlled semantic retrieval. Neuron.

[bib53] Wegner D.M. (1989). Try not to think of a white bear. Psychol. Today.

[bib54] Wegner D.M., Schneider D.J., Carter S.R., White T.L. (1987). Paradoxical effects of thought suppression. J.Pers. Soc. Psychol..

[bib55] Wenzlaff E.M., Wegner D.M. (2000). Thought suppression. Ann. Rev. Psychol..

[bib56] Williams B.R., Ponesse J.S., Schachar R.J., Logan G.D., Tannock R. (1999). Development of inhibitory control across the life span. Dev. Psychol..

